# Disinformation of text mining online about tobacco and the COVID-19 discussed on Sina Weibo

**DOI:** 10.18332/tid/142776

**Published:** 2021-10-22

**Authors:** Di Zhang, Bing Fang, Ling Yang, Yuyang Cai

**Affiliations:** 1 Department of Geriatrics, Xinhua Hospital, Shanghai Jiao Tong University School of Medicine, Shanghai, China; 2 Department of Information Management, School of Management, Shanghai University, Shanghai, China; 3 School of Medicine, Tibet University, Lhasa, China; 4 School of Public Health, Shanghai Jiao Tong University School of Medicine, Shanghai, China; 5 China Institute for Urban Governance, Shanghai Jiao Tong University, Shanghai, China

**Keywords:** tobacco, COVID-19, text mining, disinformation, new media

## Abstract

**INTRODUCTION:**

During the COVID-19 pandemic, various types of disinformation have emerged from the media. This study focuses on the online disinformation about tobacco and the COVID-19 on the Sina Weibo, the Chinese largest new media microblog platform.

**METHODS:**

The related posts from the beginning of the epidemic in December 2019 to 19 January 2021 were searched. Text mining technology was applied on these posts to identify content on ‘smoking can prevent COVID-19’. Descriptive research was used to analyze the dataset.

**RESULTS:**

Among the 912 original posts, 508 informative posts were selected after artificial recognition, including 112 posts of spreading disinformation and 396 which dispel the disinformation. Of the disinformation posts, 74% (83/112) cited the results of scientific research, and 17% (19/112) mentioned that smog from burning Asian wormwood could prevent COVID-19. By analyzing the public’s comments on these 112 disinformation posts, it was suggested that about 12% of the comments were in support, and 88% of the posts were opposed or invalid. The proportion of supportive comments on pseudo-scientific information was higher than on plain disinformation, 21% and 9%, respectively.

**CONCLUSIONS:**

The disinformation of promoting smoking as a way to prevent COVID-19 has the typical feature of using pseudo-scientific arguments to package disinformation, making it very difficult for readers without professional knowledge to identify. Such actions harm both tobacco control and COVID-19 prevention.

## INTRODUCTION

At the beginning of the COVID-19 pandemic in 2020, disinformation that smoking could prevent SARS-CoV-2 spread across China, the country with the largest number of consumers of tobacco in the world^1^. As part of COVID-19 pandemic prevention, the Chinese government took immediate action to dispel rumors and added special columns in the publication of key traditional and new media. However, even if the authorities actively refuted rumors, false information and related discussions about COVID-19 were still emerging from new media such as online forums and blogs.

While the whole world is under the series of waves of the pandemic^2^, we focused on assessing the online disinformation about tobacco and COVID-19 through text mining on Sina Weibo, the Chinese largest new media microblog platform (similar to Twitter).

## METHODS

Search strings such as ‘Nicotine, COVID-19’ and ‘Smoking, COVID-19’ were used to search the posts from the beginning of the epidemic in December 2019 to 19 January 2021. Text mining technology was applied on these posts to identify the content of ‘smoking can prevent COVID-19’. Furthermore, descriptive research was used to analyze the dataset of disinformation.

## RESULTS

As a result, 912 original posts were identified, and 508 informative posts were selected through artificial recognition. There were only 112 posts spreading disinformation and the rest (396) dispelled the disinformation.

Among the disinformation posts, only 10 posts explicitly made the statement that ‘smoking can prevent COVID-19’ and 83 posts (74%) did not present viewpoints directly, but cited the results of scientific research related to nicotine inhibiting SARS-CoV-2. Moreover, there were 19 posts mentioning that smog of burning Asian wormwood can prevent COVID-19.

**Figure 1:**
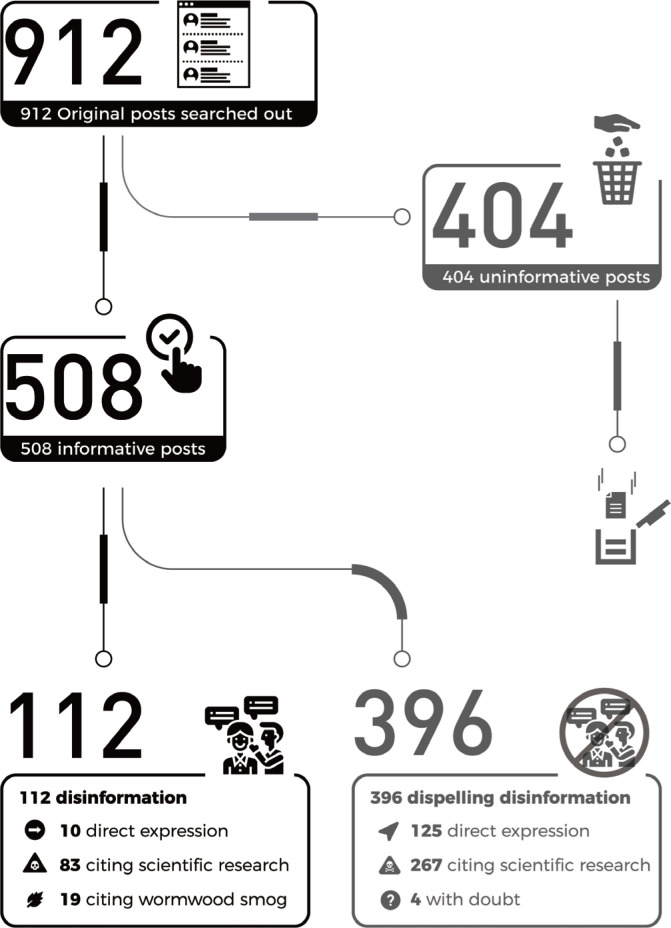
Flow of posts screening and categories

In the 396 posts that dispelled the misinformation, there were 125 posts that directly claimed that ‘smoking can prevent COVID-19’ as disinformation. A further 267 posts cited scientific knowledge or facts to contradict the disinformation, and 4 posts took a skeptical attitude towards this disinformation (Figure 1).

We further analyzed the 112 posts spreading disinformation and found that according to their different ways of expression, they can be divided into the following two categories: 1) Posts spreading disinformation that used technical terms such as nicotine and scientific research, which we call Type 1, the pseudo-scientific information; and 2) Posts using plainer statements such as ‘smoking can prevent COVID-19’, which we call Type 2, the plain disinformation.

After screening the information records, further analysis of the public’s feedback on these 112 disinformation posts was carried out by analyzing the comments. About 12% of the comments were in support, and 88% were opposed or invalid. It is worth noting that the proportion of support comments on Type 1 was higher than that for Type 2, at 21% and 9%, respectively.

## DISCUSSION

During the pandemic, the demand of the public for COVID-19 related information is increasing, in which sensational reports on social media have challenged the prevention and control of the epidemic^3^.

The statement that tobacco can prevent infectious diseases has a long history. Since the SARS period in 2003, there has been false information that smoking can prevent SARS. However, 17 years later, similar disinformation has still appeared since the initial stage of the COVID-19 pandemic. Moreover, at the initial stage in February 2020, ‘smoking can prevent COVID-19’ spread wildly on the internet in China, claiming that tobacco tar can work as a protective layer to block virus intrusion and hinder its intracellular copy by covering the surface of the alveoli.

Since the outbreak of COVID-19, the Chinese government has been making an effort to dispel the disinformation that smoking can help prevent COVID-19, and set special sections in various traditional and new media to promote the popularization of science^4^. However, the disinformation still exists, it can be seen that 74% of 112 posts cited scientific research out of context and used scientific research terms. Our results also suggest that scientifically packaged information is less detectable, more convincing and more difficult to be discovered and deleted by the relevant authorities. It is notable that almost 17% of the disinformation expressed the concept of ‘eliminating evil spirits’ in traditional Chinese culture. In traditional Chinese culture, the wormwood herb is a mascot for warding off evil spirits, and the smog of burning Asian wormwood leaves is thought to have the capability of killing bacteria and viruses. Leaf fumigation (using the smog produced by burning wormwood leaves to disinfect the environment) has been a simple method for epidemic prevention in ancient and modern China^5^.

## CONCLUSIONS

During this pandemic, disinformation does not only harm effective epidemic control, but also places a barrier to daily health behavior promotion. In China, which has more than half of the world’s smokers, spreading the disinformation that smoking can prevent COVID-19 seriously affects the effectiveness of tobacco control action. However, even if various measures have been taken to refute rumors, new media still generate confusing and misleading information. This persistent disinformation that promotes smoking has the typical feature of using pseudo-scientific arguments, making it very difficult for the ordinary citizen, without professional knowledge, to identify them. Under the influence of pseudo-scientific information, smokers have more ‘confidence’ to continue smoking, and even some will try to smoke to prevent COVID-19 infection.

Both information providers and the general public should learn how to properly evaluate the accuracy of information. This should include not being confused by the title and looking through the supporting literature, to be better informed. Healthcare professionals can play an active role in disseminating COVID-19 related scientific information to the general public to ensure that they obtain correct knowledge.

## Data Availability

The data supporting this research are available from the authors on reasonable request.
